# Supplementary material for: Contesting the presence of wheat in the British Isles 8,000 years ago by assessing ancient DNA authenticity from low-coverage data
Source: eLife. 2015 Nov 3;4:e10005. doi: 10.7554/eLife.10005 (PMC4629168; doi:10.7554/eLife.10005)
Supplement: Supplementary file 1. — Sequencing strategy. DOI: http://dx.doi.org/10.7554/eLife.10005.009 [file elife10005s001.docx]

**Supplementary File 1.** Sequencing strategy

| Sample ID | Type of sequencing read | Read length^**^ |
| --- | --- | --- |
| ERR567364^*^ | Single end | 256 |
| ERR567365^*^ | Single end | 256 |
| ERR567366^*^ | Single end | 256 |
| ERR567367^*^ | Single end | 256 |
| ERR732642^*^ | Single end | 256 |
| SRR1170664^*^ | Single end | 256 |
| ERR267886^*^ | Paired end | 101 |
| ERR267882^*^ | Paired end | 151 |
| ERR330058^*^ | Paired end | 101 |
| ERR267872^*^ | Paired end | 151 |
| ERR267868^*^ | Paired end | 151 |
| ERR957324^*^ | Paired end | 151 |
| ERR267866^*^ | Paired end | 151 |
| ERR267884^*^ | Paired end | 101 |
| ERR267878^*^ | Paired end | 101 |
| 107^+^ | Paired end | 101 |
| 109^+^ | Paired end | 101 |
| 110^+^ | Paired end | 101 |
| 114^+^ | Paired end | 101 |
| SRR1045127 | Single end | 94 |

^*^IDs from the European Nucleotide Archive as in Table 1; IDs from Sawyer *et al*. 2012, as in Table 1; ^**^Length in base pairs
